# *RET* Variants and Haplotype Analysis in a Cohort of Czech Patients with Hirschsprung Disease

**DOI:** 10.1371/journal.pone.0098957

**Published:** 2014-06-04

**Authors:** Eliska Vaclavikova, Sarka Dvorakova, Richard Skaba, Lucie Pos, Vlasta Sykorova, Tereza Halkova, Josef Vcelak, Bela Bendlova

**Affiliations:** 1 Department of Molecular Endocrinology, Institute of Endocrinology, Prague, Czech Republic; 2 Department of Paediatric Surgery, 2nd Faculty of Medicine, Charles University and Hospital Motol, Prague, Czech Republic; MOE Key Laboratory of Environment and Health, School of Public Health, Tongji Medical College, Huazhong University of Science and Technology, China

## Abstract

Hirschsprung disease (HSCR) is a congenital aganglionosis of myenteric and submucosal plexuses in variable length of the intestine. This study investigated the influence and a possible modifying function of *RET* proto-oncogene's single nucleotide polymorphisms (SNPs) and haplotypes in the development and phenotype of the disease in Czech patients. Genotyping of 14 SNPs was performed using TaqMan Genotyping Assays and direct sequencing. The frequencies of SNPs and generated haplotypes were statistically evaluated using chi-square test and the association with the risk of HSCR was estimated by odds ratio. SNP analysis revealed significant differences in frequencies of 11 polymorphic *RET* variants between 162 HSCR patients and 205 unaffected controls. Particularly variant alleles of rs1864410, rs2435357, rs2506004 (intron 1), rs1800858 (exon 2), rs1800861 (exon 13), and rs2565200 (intron 19) were strongly associated with increased risk of HSCR (p<0.00000) and were over-represented in males vs. females. Conversely, variant alleles of rs1800860, rs1799939 and rs1800863 (exons 7, 11, 15) had a protective role. The haploblock comprising variants in intron 1 and exon 2 was constructed. It represented a high risk of HSCR, however, the influence of other variants was also found after pruning from effect of this haploblock. Clustering patients according to genotype status in haploblock revealed a strong co-segregation with several SNPs and pointed out the differences between long and short form of HSCR. This study involved a large number of SNPs along the entire *RET* proto-oncogene with demonstration of their risk/protective role also in haplotype and diplotype analysis in the Czech population. The influence of some variant alleles on the aggressiveness of the disease and their role in gender manifestation differences was found. These data contribute to worldwide knowledge of the genetics of HSCR.

## Introduction

Hirschsprung disease (HSCR) is a congenital developmental malformation characterised by the absence of enteric ganglion cells of myenteric and submucosal plexuses in the intestine. The incidence of the disease is 1 per 5000 live births. Short-segment aganglionosis (80%) and long-segment aganglionosis (20%) are classified according to the length of the aganglionic segment. The short-segment form of HSCR (S-HSCR), comprising recto sigmoid HSCR and ultra-short segment HSCR, affects the distal portion, part of the anal canal and, in contrast to long-segment HSCR, occurs four times more often in males than females. The long-segment form of HSCR (L-HSCR) can present as total colonic aganglionosis (TCA) extending from the rectum up to the terminal ileum and, in rare cases, as nearly total bowel (NTBA) or total intestinal aganglionosis (TIA) comprising nearly total or the whole intestine. It occurs as an isolated disorder in 70% of cases, about 12% of cases have a chromosomal abnormality and 18% of cases have additional congenital anomalies [1].

Isolated HSCR appears as a sporadic (85%) or less commonly as a familial disorder (15%). To date, more than 14 genes and 5 susceptibility loci have been associated with the disease [1], [2]. However, the inactivating germline mutations in the *RET* proto-oncogene play the major role in the pathogenesis. The *RET* (Rearranged during Transfection), a gene consisting of 21 exons and located on chromosome 10q11.2, encodes a transmembrane tyrosine kinase receptor. It is expressed in neural crest-derived cells and plays an important role in the development, proliferation and differentiation of neuroendocrine cells. The various alterations (missense mutations, deletions, insertions, frame shifts) have been found along the entire gene. Inactivating germline *RET* mutations are detected in about 50% of familial and 15–20% of sporadic HSCR cases [1].

Although the detection rate of germline *RET* mutations is relatively low, linkage analysis has shown that nearly all familial HSCR patients are in linkage with the *RET* proto-oncogene [3]. Therefore, noncoding variants and single nucleotide polymorphisms (SNPs) as well as some specific haplotypes of the *RET* proto-oncogene have been revealed to be a potential low susceptibility loci and have a function of genetic modifying factors in HSCR pathogenesis. Several *RET* polymorphisms have been investigated in the association with HSCR. Previous studies [3]–[5] described the variants in the coding regions - polymorphism in exon 2 (rs1800858, p.Ala45Ala, c.135G/A) was overrepresented in HSCR patients with respect to controls, as well as polymorphisms in exon 7 (rs1800860, p.Ala432Ala, c.1296G/A) and in exon 13 (rs1800861, p.Leu769Leu, c.2307T/G) are risk factors for HSCR. On the other hand, SNPs in exons 11 (rs1799939, p.Gly691Ser, c.2071G/A), 14 (rs1800862, p.Ser836Ser, c.2508C/T) and 15 (rs1800863, p.Ser904Ser, c.2712C/G) were underrepresented in HSCR cases, suggesting a protective role of these SNPs. However, results could differ depending on methods, cohorts and frequencies of SNPs.

Consequently, the study of SNPs has been focused on noncoding regions - 5′untranslated region (UTR), intron variants and 3′UTR. The investigation of the strong association of rs1800858 (p.Ala45Ala, c.135G/A) with HSCR was extended upstream of exon 2 to intron 1 and promoter. A haplotype spanning 27 kb of the 5′UTR region to exon 2 was determined as HSCR-associated [2], [6]–[8]. The investigation of 3′UTR variants underrepresented in HSCR patients resulted in the suggestion of the protective haplotype in 3′UTR [9]–[10].

In this large case-control study, we investigated the possible role of variants in the *RET* proto-oncogene in cohorts of 162 Czech patients with HSCR and 205 unaffected control individuals and the role of these variants in gender manifestation differences and their influence on the aggressiveness of the disease. We carried out a screening of 14 polymorphisms throughout the *RET* proto-oncogene to identify risk or protective SNPs and haplotypes associated with the HSCR phenotype from the Czech population.

## Material and Methods

In this study, blood samples were obtained from 162 Czech patients with HSCR (121 males and 41 females). The patients were chosen for molecular genetic analysis prospectively (88 cases operated on from 2003 till now) as well as retrospectively (74 cases operated on between 1979 and 2003). According to the length of the aganglionic segment, the cohort consisted of 117 patients with short-segment HSCR (recto-sigmoid form; patients with ultra-short segment were not involved in the study), 41 patients with long-segment aganglionosis (including 20 patients with total colonic aganglionosis and 2 patients with nearly total bowel aganglionosis), and 4 patients with an unspecified form of HSCR. The control group included 205 Czech healthy individuals (95 males, 110 females).

### Ethics statement

This study was approved by the Ethical Committee of the Institute of Endocrinology and University Hospital Motol, Prague. A signed informed consent for this study was obtained from each patient or legitimate representative who participated.

### Genetic analysis

Genomic DNA was isolated from peripheral blood leukocytes using the QIAamp DNA Blood Kit (Qiagen, Germany) and the QuickGene 610 L machine (Fujifilm, Japan). Studied polymorphisms are listed in Table 1. Five of SNPs were analysed during routine sequencing testing of HSCR patients for mutations in exons 10, 11, 13, 14, 15 and 16 in the *RET* proto-oncogene due to the risk of medullary thyroid cancer. The standard conditions for amplification of *RET* exons were provided previously [11] as well as sequenase reaction preparation [12]. Direct sequencing was performed on the CEQ 8000 sequencing machine (Beckman Coulter) and analysed by CEQ 8000 sequencing software. Sequence traces were compared with *RET* reference sequence (NG_007489.1). Genotyping of the other 9 SNPs in patients and all 14 SNPs in control samples were determined by real-time polymerase chain reaction (PCR) with TaqMan Genotyping Assays (Applied Biosystems) on LightCycler 480 Real-Time PCR System (Roche) with using non template control in each run.

**Table 1:** Allelic distribution of single nucleotide polymorphisms in HSCR patients and controls.

SNP	Location	Amino acid change	Nucleotide change	HSCR (n = 162): Variant allele (%)	Controls (n = 205): Variant allele (%)	p-value (for χ^2^)	OR (95% CI)	p-value (for OR)
rs1864410	Intron 1	IVS1+2846G/T	c.73+2846G/T	233 (71.9)	113 (27.6)	**0.00000**	6.73 (4.86–9.31)	**0.00000**
rs2435357	Intron 1	IVS1+9277C/T	c.73+9277C/T	234 (72.2)	115 (28.0)	**0.00000**	6.67 (4.82–9.23)	**0.00000**
rs2506004	Intron 1	IVS1+9494C/A	c.73+9494C/A	234 (72.2)	115 (28.0)	**0.00000**	6.67 (4.82–9.23)	**0.00000**
rs1800858	Exon 2	Ala45Ala	c.135G/A	231 (71.3)	112 (27.3)	**0.00000**	6.61 (4.78–9.14)	**0.00000**
rs1800860	Exon 7	Ala432Ala	c.1296G/A	82 (25.8)	139 (33.9)	**0.01816**	0.68 (0.49–0.94)	**0.02255**
rs1799939	Exon 11	Gly691Ser	c.2071G/A	27 (8.3)	94 (22.9)	**0.00000**	0.31 (0.19–0.48)	**0.00000**
rs1800861	Exon 13	Leu769Leu	c.2307T/G	141 (43.5)	89 (21.8)	**0.00000**	2.76 (2.00–3.81)	**0.00000**
rs111264957	Intron 13	IVS13-94C/T	c.2393-94C/T	6 (1.9)	12 (2.9)	0.34978	0.63 (0.23–1.69)	0.48723
rs1800862	Exon 14	Ser836Ser	c.2508C/T	8 (2.5)	12 (2.9)	0.70528	0.84 (0.34–2.08)	0.88083
rs2472737	Intron 14	IVS14-24G/A	c.2608-24G/A	82 (25.3)	99 (24.1)	0.71678	1.06 (0.76–1.49)	0.78213
rs1800863	Exon 15	Ser904Ser	c.2712C/G	28 (8.6)	94 (22.9)	**0.00000**	0.32 (0.20–0.50)	**0.00000**
rs2565200	Intron 19	IVS19-627C/T	c.3188-627C/T	133 (41.0)	77 (18.8)	**0.00000**	3.01 (2.16–4.20)	**0.00000**
rs143948954	3′UTR		c.4391G/C	8 (2.5)	2 (0.5)	**0.02149**	5.16 (1.09–24.49)	**0.04785**
rs2435355	3′UTR		c.4461T/C	105 (32.6)	103 (25.1)	**0.02579**	1.44 (1.04–1.99)	**0.03181**

### Statistical analysis

The frequencies of studied SNPs were statistically evaluated and compared using the NCSS programme and chi-square test with establishing p-value. The results were considered as statistically significant if the p-value was less than 0.05. Association with risk of HSCR was estimated by odds ratio (OR) and their 95% confidence interval (CI). For generating haplotypes, the Haploview programme (version 4.1) was used and a haplotype block of polymorphisms which were in linkage disequilibrium was constructed using Gabriel's methods. Consequently, haplotypes of the particular haploblock were generated in each individual patient and control and diplotypes were estimated using the PHASE programme (version 2.1).

## Results

### SNP analysis

Statistical evaluation of studied SNPs revealed significant differences in genotype and allele distribution of polymorphic variants between HSCR patients and normal controls (Table 1). No significant deviation from Hardy-Weinberg equilibrium was found in the control cohort. Studied cohorts significantly differed in 11 out of 14 investigated SNPs. In SNPs rs1864410, rs2435357, rs2506004, rs1800858 the variant allele dominated in HSCR patients (all p<0.00000) and their carriership was associated with more than 6.6-fold elevated risk for development of HSCR compared with the wild-type allele carriership. These 4 SNPs were in complete linkage disequilibrium and went together with almost the same genotype distribution in all cohorts. The variant allele was also over-represented in rs1800861 (p<0.00000, OR = 2.76, 95% CI = 2.00–3.81) and rs2565200 (p<0.00000, OR = 3.01, 95% CI = 2.16–4.20). In contrast, the variant allele was under-represented in two SNPs in linkage disequilibrium: rs1799939 (p<0.00000, OR = 0.31, 95% CI = 0.19–0.48) and rs1800863 (p<0.00000, OR = 0.32, 95% CI = 0.20–0.50). Significant differences in allele distribution were established in rs1800860, rs143948954 and rs2435355. Allele distributions of SNPs considering the phenotype of the disease (L- and S-HSCR) and gender were described in Table S1 and Table S2.

Three SNPs (rs111264957, rs1800862, rs143948954) were detected in very low frequencies of variant allele (2.9%, 2.9%, and 0.5%, respectively in controls).

### Haplotype and diplotype analysis

**Figure 1:**
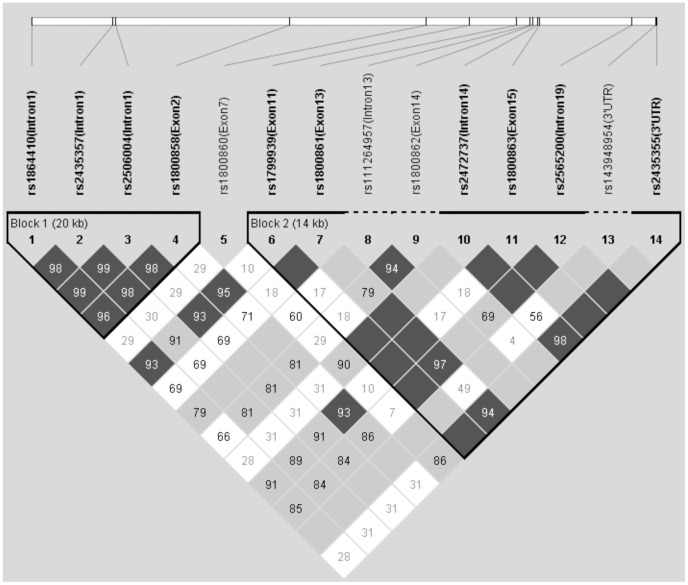
Haplotype blocks generated by the Haploview Programme in cohorts of HSCR patients and control population. The scheme is shown with confidence bounds. LD values are reported in D′.

Using the Haploview programme for generating haplotypes, two haplotype blocks were identified (Figure 1) comprising 10 out of 14 investigated polymorphisms that were in linkage disequilibrium. The first haploblock was composed of 4 SNPs (rs1864410, rs2435357, rs2506004, rs1800858) that were very closely related together. The second haploblock consisting of 6 SNPs (rs1799939, rs1800861, rs2472737, rs1800863, rs2565200, rs2435355) was very heterogeneous and differed in distribution between particular cohorts. Therefore, these SNPs were rather evaluated separately. Moreover, 4 SNPs not included in any of two haploblocks due to low frequencies in cohorts or its localization in the gene were also investigated separately.

The two most frequent haplotypes of 5′ region haploblock were represented by TTAA and GCCG (Table 2). The presence of only two main haplotypes was caused by above-mentioned trend that in nearly all cases these 4 SNPs went together in wild type or variant allele mode. The distribution of haploblock-haplotypes significantly differed between HSCR patients and controls (p<0.00000). Although the most frequent haplotype in HSCR patients was TTAA (HSCR 70.7% vs. controls 26.1%), the most frequent in controls was haplotype GCCG (HSCR 27.5% vs. controls 71.2%). The haplotype TTAA had a 6.83-fold (95% CI = 4.93–9.45, p<0.00000) elevated risk for development of HSCR than other haplotypes. The different distribution of estimated combinations of haplotypes in two alleles of each subject resulted from this converse representation of haplotypes. The diplotype TTAA,TTAA had a 17,56-fold (95% CI = 9.25–33.35, p<0.00000) elevated risk for the development of HSCR than other diplotypes.

**Table 2:** Distribution of haplotypes and diplotypes of 5′ region haploblock in HSCR patients and controls.

Haplotypes of haploblock*		HSCR (%)	Controls (%)	p-value (for χ^2^)	OR (95% CI)	p-value (for OR)
1	TTAA	229 (70.7)	107 (26.1)	**0.00000**	6.83 (4.93–9.45)	**0.00000**
2	GCCG	89 (27.5)	292 (71.2)	**0.00000**	0.15 (0.11–0.21)	**0.00000**

Diplotypes of haploblock*		HSCR (%)	Controls (%)	p-value (for χ^2^)	OR (95% CI)	p-value (for OR)
1,1	TTAA,TTAA	88 (54.3)	13 (6.3)	**0.00000**	17.56 (9.25–33.35)	**0.00000**
1,2	TTAA,GCCG	50 (30.9)	75 (36.6)	0.25081	0.77 (0.50–1.20)	0.29951
2,2	GCCG,GCCG	18 (11.1)	106 (51.7)	**0.00000**	0.12 (0.07–0.20)	**0.00000**

* Haplotypes/diplotypes with occurrence <2% in both cohorts are not included.

If we consider the phenotype of the disease with haploblock status, variant allele frequencies in L-HSCR and S-HSCR were significantly different from controls, but did not vary between forms of HSCR (Table 3). The risk of HSCR was higher in the S-form than in L-form resulting from odds ratios (7.62 vs. 5.66 for rs1864410). By comparing frequencies of SNPs in haploblock between male patients and male controls as well as female patients and female controls, similar significant results were obtained–contrary representation of the variant allele in controls vs. patients. In comparison between male and female patients, there were also statistically significant differences. The different allele distribution in haploblock (for rs1864410 variant allele in male 76.0% vs. female 59.8%) was related to the different risk for development of HSCR in males (OR = 8.20, 95% CI = 5.32–12.65) and females (OR = 3.96, 95% CI = 2.33–6.74).

**Table 3:** Allelic distribution of single nucleotide polymorphisms of 5′ region haploblock in patients with long-segment and short-segment form of HSCR and in male and female HSCR patients.

	L-HSCR (n = 41)					S-HSCR (n = 117)					L-HSCR vs. S-HSCR		
SNP	Cases: Variant allele (%)	Controls: Variant allele (%)	p-value (for χ^2^)	OR (95% CI)	p-value (for OR)	Cases: Variant allele (%)	Controls: Variant allele (%)	p-value (for χ^2^)	OR (95% CI)	p-value (for OR)	p-value (for χ^2^)	OR (95% CI)	p-value (for OR)
rs1864410	56 (68.3)	113 (27.6)	**0.00000**	5.66 (3.39–9.46)	**0.00000**	174 (74.4)	113 (27.6)	**0.00000**	7.62 (5.29–10.98)	**0.00000**	0.28819	0.74 (0.43–1.29)	0.35865
rs2435357	57 (69.5)	115 (28.0)	**0.00000**	5.85 (3.49–9.81)	**0.00000**	174 (74.4)	115 (28.0)	**0.00000**	7.44 (5.17–10.71)	**0.00000**	0.39437	0.79 (0.45–1.37)	0.47956
rs2506004	57 (69.5)	115 (28.0)	**0.00000**	5.85 (3.49–9.81)	**0.00000**	174 (74.4)	115 (28.0)	**0.00000**	7.44 (5.17–10.71)	**0.00000**	0.39437	0.79 (0.45–1.37)	0.47956
rs1800858	55 (67.1)	112 (27.3)	**0.00000**	5.42 (3.26–9.02)	**0.00000**	173 (73.9)	112 (27.3)	**0.00000**	7.55 (5.24–10.86)	**0.00000**	0.23315	0.72 (0.42–1.24)	0.29412

	Male cases (n = 121) vs. male controls (n = 95)					Female cases (n = 41) vs. female controls (n = 110)					Male vs. female cases		
SNP	Cases: Variant allele (%)	Controls: Variant allele (%)	p-value (for χ^2^)	OR (95% CI)	p-value (for OR)	Cases: Variant allele (%)	Controls: Variant allele (%)	p-value (for χ^2^)	OR (95% CI)	p-value (for OR)	p-value (for χ^2^)	OR (95% CI)	p-value (for OR)
rs1864410	184 (76.0)	53 (27.9)	**0.00000**	8.20 (5.32–12.65)	**0.00000**	49 (59.8)	60 (27.3)	**0.00000**	3.96 (2.33–6.74)	**0.00000**	**0.00459**	2.14 (1.26–3.63)	**0.00710**
rs2435357	185 (76.4)	54 (28.4)	**0.00000**	8.17 (5.30–12.60)	**0.00000**	49 (59.8)	61 (27.7)	**0.00000**	3.87 (2.28–6.58)	**0.00000**	**0.00354**	2.19 (1.28–3.72)	**0.00554**
rs2506004	185 (76.4)	54 (28.4)	**0.00000**	8.17 (5.30–12.60)	**0.00000**	49 (59.8)	61 (27.7)	**0.00000**	3.87 (2.28–6.58)	**0.00000**	**0.00354**	2.19 (1.28–3.72)	**0.00554**
rs1800858	181 (74.8)	53 (27.9)	**0.00000**	7.67 (4.99–11.79)	**0.00000**	50 (61.0)	59 (26.8)	**0.00000**	4.26 (2.50–7.28)	**0.00000**	**0.01683**	1.90 (1.12–3.23)	**0.02450**

### Distribution of 3′ region SNPs with respect to 5′ region haploblock status

Other SNPs located in 3′ *RET* region were examined depending on what haplotype in 5′ part of gene was carried. Given allelic distribution of TTAA and GCCG haplotypes (Table 4), frequencies of variant alleles of rs1800861 and 2565200 as well as rs1799939 and rs1800863 significantly differed between cases and controls with the same risk/protective trends as were shown in previous SNP analysis (Table 1). In genotype distribution of these haplotypes, there was possible to observe the tendency of the increasing/decreasing variant allele frequencies depending on carriership of TTAA in two, one or no alleles (Table S3).

**Table 4:** Allelic distribution of single nucleotide polymorphisms considering allelic distribution of haploblock-haplotypes TTAA and GCCG in HSCR patients and controls.

	TTAA: 70.7% cases (88 homozygotes; 50 heterozygotes) vs. 26.1% controls (13 homozygotes; 75 heterozygotes)				GCCG: 27.5% cases (18 homozygotes; 50 heterozygotes) vs. 71.2% controls (106 homozygotes; 75 heterozygotes)			
SNP	Cases: Variant allele (%)	Controls: Variant allele (%)	OR (95% CI)	p-value	Cases: Variant allele (%)	Controls: Variant allele (%)	OR (95% CI)	p-value
rs1800860	108 (24.2)	50 (24.8)	0.97 (0.66–1.43)	0.96110	54 (32.5)	216 (37.6)	0.80 (0.55–1.15)	0.26667
rs1799939	15 (3.3)	19 (9.4)	0.33 (0.16–0.66)	**0.00229**	35 (20.4)	161 (28.1)	0.66 (0.43–0.99)	0.05563
rs1800861	233 (51.6)	78 (38.6)	1.69 (1.21–2.37)	**0.00292**	45 (26.2)	86 (15.0)	2.01 (1.33–3.03)	**0.00109**
rs111264957	4 (0.9)	3 (1.5)	0.59 (0.13–2.67)	0.78107	8 (4.7)	19 (3.3)	1.42 (0.61–3.31)	0.55297
rs1800862	6 (1.3)	3 (1.5)	0.89 (0.22–3.60)	0.83892	10 (5.8)	19 (3.3)	1.80 (0.82–3.96)	0.20575
rs2472737	129 (28.5)	63 (31.2)	0.88 (0.61–1.26)	0.55238	27 (15.7)	119 (20.7)	0.71 (0.45–1.13)	0.17698
rs1800863	17 (3.8)	19 (9.4)	0.38 (0.19–0.74)	**0.00616**	35 (20.4)	161 (28.1)	0.66 (0.43–0.99)	0.05563
rs2565200	226 (50.0)	76 (37.6)	1.66 (1.18–2.33)	**0.00439**	36 (20.9)	64 (11.2)	2.11 (1.34–3.31)	**0.00150**
rs143948954	14 (3.1)	3 (1.5)	2.12 (0.60–7.46)	0.35174	2 (1.2)	1 (0.2)	6.74 (0.61–74.80)	0.26690
rs2435355	160 (35.6)	61 (30.2)	1.28 (0.89–1.82)	0.21242	40 (23.5)	129 (22.5)	1.06 (0.71–1.59)	0.85377

In carriers of TTAA haplotype, differences between L-HSCR and S-HSCR phenotype were intensified in variant alleles of rs1800861, rs2472737, rs2565200 and rs2435355, whereas in carriers of GCCG no significant differences between phenotypes were found (Table 5). In TTAA carriers, the variant alleles of rs1800861 and rs2565200 were over-represented in S-HSCR (55.6% and 54.1%, respectively) vs. L-HSCR (37.7% and 36.8%, respectively). The variant alleles of rs2472737 and rs2435355 were over-represented in L-HSCR (39.6% and 44.2%, respectively) vs. S-HSCR (25.0% and 32.9%, respectively). Genotype distribution of haploblock-haplotypes TTAA and GCCG describing differences in 3′ region SNPs frequencies between L- and S-HSCR is shown in Table S4. No differences between male and female patients and controls were observed considering the 5′ region haplotype (Table S5).

**Table 5:** Allelic distribution of single nucleotide polymorphisms considering allelic distribution of haploblock-haplotypes TTAA and GCCG in patients with long-segment and short-segment form of HSCR.

	TTAA: 64.6% L-HSCR (n = 19 homozygotes; 15 heterozygotes) vs. 72.6% S-HSCR (n = 68 homozygotes; 34 heterozygotes)				GCCG: 28.0% L-HSCR (n = 4 homozygotes; 15 heterozygotes) vs. 24.8% S-HSCR (n = 12 homozygotes; 34 heterozygotes)			
SNP	L-HSCR: Variant allele (%)	S-HSCR: Variant allele (%)	OR (95% CI)	p-value	L-HSCR: Variant allele (%)	S-HSCR: Variant allele (%)	OR (95% CI)	p-value
rs1800860	19 (17.9)	89 (26.7)	0.60 (0.35–1.04)	0.09132	15 (32.6)	39 (34.2)	0.93 (0.45–1.93)	0.99263
rs1799939	5 (4.7)	10 (2.9)	1.63 (0.55–4.89)	0.56398	9 (19.6)	24 (20.7)	0.93 (0.40–2.19)	0.95528
rs1800861	40 (37.7)	189 (55.6)	0.48 (0.31–0.76)	**0.00194**	14 (30.4)	27 (23.3)	1.44 (0.67–3.09)	0.45650
rs111264957	2 (1.9)	1 (0.3)	6.52 (0.59–72.62)	0.28414	4 (8.7)	3 (2.6)	3.59 (0.77–16.71)	0.19498
rs1800862	3 (2.8)	2 (0.6)	4.92 (0.81–29.86)	0.16578	5 (10.9)	4 (3.4)	3.41 (0.87–13.34)	0.13912
rs2472737	42 (39.6)	85 (25.0)	1.97 (1.24–3.12)	**0.00528**	10 (21.7)	17 (14.7)	1.62 (0.68–3.86)	0.39136
rs1800863	5 (4.7)	12 (3.5)	1.35 (0.47–3.93)	0.78944	9 (19.6)	24 (20.7)	0.93 (0.40–2.19)	0.95528
rs2565200	39 (36.8)	184 (54.1)	0.49 (0.32–0.77)	**0.00267**	9 (19.6)	24 (20.7)	0.93 (0.40–2.19)	0.95528
rs143948954	2 (1.9)	12 (3.5)	0.53 (0.12–2.39)	0.59762	0 (0.0)	2 (1.7)	0.82 (0.08–8.04)	0.70147
rs2435355	46 (44.2)	112 (32.9)	1.61 (1.03–2.53)	**0.04689**	12 (27.3)	28 (24.1)	1.18 (0.54–2.59)	0.83801

## Discussion

The genetics of HSCR is complex. It is believed that it can be inherited in a dominant or recessive trait, but probably it is polygenic with incomplete penetrance, genetic heterogeneity, variable expression of the disease and a large number of additive syndromes. The main impact of the *RET* proto-oncogene mutation is a loss of function (haploinsufficiency). Almost all HSCR cases are linked to the *RET* locus despite no detected mutation. Therefore, noncoding *RET* variants and SNPs must play at least a modifying role in the remaining HSCR cases.

We focused our study on SNPs along the entire *RET* proto-oncogene and selected 14 promising SNPs in coding and intronic sequences and 3′UTR. Allele frequencies in our tested normal population were similar with frequencies in previously reported in a European control population [13] and greatly varied from an Asian control population [2], [10], [14], [15].

Our data show that mainly 8 SNPs are very important in the development of HSCR. We found a strong association of variant alleles of 4 investigated SNPs in intron 1 and exon 2 grouped in haploblock (rs1864410, rs2435357, rs2506004, rs1800858) with HSCR (p<0.00000). These alleles were the most frequent in our patient cohort and formed the haplotype TTAA with a very elevated risk (OR = 6.83). In the homozygous diplotype TTAA,TTAA the risk was even more increased (OR = 17.56). These findings are consistent with other studies describing these SNPs as belonging to HSCR-causing region covering 27 kb in total. It is a highly conserved region called MSC+9.7 (Multi species conserved) and starts 4 kb upstream of the *RET* transcription start site and going along the way to the beginning of exon 2. This risk haplotype decreases *RET* promoter activity, reduces the binding affinity of TTF-1 (thyroid transcription factor 1), disrupts a binding site for transcription factor SOX10, decreases enhancer for RET expression and thus regulates RET expression which was confirmed by in vitro studies [7], [8], [16], [17].

In previous studies, there was suggested the hypothesis about the different role of SNPs in two linkage disequilibrium regions. Besides the risk haplotype at the 5′ end of the *RET* proto-oncogene, a protective function of the 3′ half of the gene was proposed. SNPs in this region encompassing rs1799939 up to 3′UTR variants were reported as underrepresented in HSCR patients [3], [9], [10]. We cannot confirm this hypothesis because we detected several SNPs with increased risk in this region. The main contradiction with the hypothesis of the protective 3′ half *RET* gene haplotype is the high overrepresentation of rs2565200 located in intron 19 and rs2435355 located in 3′UTR. SNP rs1800861 in exon 13 was also strongly associated with HSCR as previously described [3], [15]. The risk effect of rs1800861 and rs2565200 in Czech patients was confirmed. Moreover, after pruning from the effect of 5′ region haploblock, variant allele frequencies of rs1800861 and rs2565200 still significantly differed in comparison of patients and controls and were associated with the risk of HSCR. In rs2435355, a divergent tendency was observed in Czech patients that differed from a study of Chinese patients [10] which instead had a protective role. Perhaps the discrepancy in the theory of protective 3′ half gene role can be due to various studied cohorts and ethnic differences. We defined three main protective SNPs in our series located in exons 7 (rs1800860), 11 (rs1799939) and 15 (rs1800863). The protective role of rs1799939 and rs1800863 was described previously [3]. Considering the 5′ region haploblock status in investigated carriers, only rs1799939 with rs1800863 variant alleles stayed protective. In contrast to our results, some studies detected rs1800862 variant allele underrepresented in HSCR with a protective role [18], and in the Chinese population the variant allele was even absent [14], which was correlated with increased HSCR incidence in the Chinese population. We identified this SNP in similar low frequencies in both our case and control cohorts, which was not in agreement with these findings. It seems that the other SNPs studied do not have any major role in the development of HSCR in our series. The low frequencies of variant allele were also identified in rs111264957 that was in linkage disequilibrium with rs1800862, and rs143948954, where a significantly higher representation of the variant allele in patients was noticed. However, due to very low frequency, this significance on the phenotype could not be definitively revealed.

In our study, we also focused on the evaluation of the possible influence of the variant alleles on aggressiveness of the disease (L-HSCR vs. S-HSCR) and their role in gender manifestation differences. The risk of variant alleles in the 5′ region haploblock was highly elevated both in L-HSCR and S-HSCR patients in relation to controls. Interestingly, the frequency of these risk alleles was even 6% higher in the less aggressive S-HSCR form. Similar results were described in rs2435357 [8] and rs1800858 [5], whereas no difference was revealed between L-HSCR and S-HSCR by Lantieri et al [3]. However, in our cohort, the higher frequency of variant alleles in S-HSCR was likely caused by a higher representation of male patients in S-HSCR (78%) than in the L-HSCR cohort (71%). A gender effect was previously described in rs2435357 where the variant allele was present in 65% of males vs. 56% of females [19]. Our data documented this trend not only in SNPs of the haploblock (76% of males vs. 60% of females) but also in risk variant alleles of rs1800861 and rs2565200. The frequency of these two risk alleles was about 10% higher in males, but nonsignificant. In variants of the haploblock, the associated risk was much more profound (OR = 8 in males vs. OR = 4 in females). It is not clear if over-representation of rs1800861 and rs2565200 in S-HSCR patients and a significant difference related to L-HSCR was also influenced by sex differences between cohorts. The risk of L-HSCR was influenced especially by rs2472737 and rs2435355 where the variant allele elevated the risk of L-HSCR nearly two fold compared to S-HSCR. Both these findings–rs1800861 and rs2565200 variant allele over-representation in S-HSCR and rs2472737 and rs2435355 variant allele over-representation in L-HSCR–were even confirmed considering the 5′ region haploblock status, but only in TTAA carriers.

## Conclusions

This study aimed to identify the risk and protective SNPs and haplotypes that could be associated with Hirschsprung disease. From our results, we propose several risk and several protective SNPs and haplotypes. The influence of some variant alleles on the aggressiveness of the disease and differences in allele frequencies between males and females were confirmed. These data could contribute to world data about the genetics of HSCR because we studied a large collection of SNPs in the *RET* proto-oncogene. We did not confirm the hypothesis about some protective SNPs in 3′UTR *RET* region. It seems that it would be more difficult and it depends on the exact SNPs and their position and function in the gene. The molecular function background of all these SNPs needs to be elucidated and the connection between SNPs among one gene seems also to be important and interesting.

## Supporting Information

Table S1 Allelic distribution of single nucleotide polymorphisms in patients with long-segment and short-segment form of HSCR.
(DOC)

Table S2 Allelic distribution of single nucleotide polymorphisms in male and female HSCR patients.
(DOC)

Table S3 Allelic distribution of single nucleotide polymorphisms considering genotypic distribution of haploblock-haplotypes TTAA and GCCG in HSCR patients and controls.
(DOC)

Table S4 Allelic distribution of single nucleotide polymorphisms considering genotypic distribution haploblock-haplotypes TTAA and GCCG in patients with long-segment and short-segment form of HSCR.
(DOC)

Table S5 Allelic distribution of single nucleotide polymorphisms considering haplotype TTAA in male and female HSCR patients.
(DOC)
