# Supplementary material for: RET Variants and Haplotype Analysis in a Cohort of Czech Patients with Hirschsprung Disease
Source: PLoS One. 2014 Jun 4;9(6):e98957. doi: 10.1371/journal.pone.0098957 (PMC4045806; doi:10.1371/journal.pone.0098957)
Supplement: Table S1 — Allelic distribution of single nucleotide polymorphisms in patients with long-segment and short-segment form of HSCR. (DOC) [file pone.0098957.s001.doc]

**Table S1** Allelic distribution of single nucleotide polymorphisms in patients with long-segment and short-segment form of HSCR

|  | **L-HSCR** (n=41) | | **S-HSCR** (n=117) | | **L-HSCR vs. S-HSCR** | | |
| --- | --- | --- | --- | --- | --- | --- | --- |
| **SNP** | **Variant allele (%)** | **p-value (for χ^2^) vs. controls** | **Variant allele (%)** | **p-value (for χ^2^) vs. controls** | **p-value (for χ^2^) L- vs. S-HSCR** | **OR (95% CI)** | **p-value (for OR)** |
| rs1864410 | 56 (68.3) | **0.00000** | 174 (74.4) | **0.00000** | 0.28819 | 0.74 (0.43 – 1.29) | 0.35865 |
| rs2435357 | 57 (69.5) | **0.00000** | 174 (74.4) | **0.00000** | 0.39437 | 0.79 (0.45 – 1.37) | 0.47956 |
| rs2506004 | 57 (69.5) | **0.00000** | 174 (74.4) | **0.00000** | 0.39437 | 0.79 (0.45 – 1.37) | 0.47956 |
| rs1800858 | 55 (67.1) | **0.00000** | 173 (73.9) | **0.00000** | 0.23315 | 0.72 (0.42 – 1.24) | 0.29412 |
| rs1800860 | 17 (20.7) | **0.01930** | 65 (28.3) | 0.14168 | 0.18355 | 0.66 (0.36 – 1.22) | 0.23649 |
| rs1799939 | 9 (11.0) | **0.01517** | 17 (7.3) | **0.00000** | 0.29268 | 1.57 (0.67 – 3.68) | 0.41293 |
| rs1800861 | 27 (32.9) | **0.03075** | 110 (47.0) | **0.00000** | **0.02681** | 0.55 (0.33 – 0.94) | **0.03709** |
| rs111264957 | 3 (3.7) | 0.72498 | 2 (0.8) | 0.08286 | 0.07997 | 4.41 (0.72 – 26.85) | 0.21622 |
| rs1800862 | 4 (4.9) | 0.36317 | 3 (1.3) | 0.18319 | 0.05692 | 3.95 (0.86 – 18.03) | 0.14212 |
| rs2472737 | 29 (35.4) | **0.03452** | 52 (22.2) | 0.57936 | **0.01898** | 1.92 (1.11 – 3.31) | **0.02789** |
| rs1800863 | 9 (11.0) | **0.01517** | 18 (7.7) | **0.00000** | 0.36006 | 1.48 (0.64 – 3.44) | 0.49290 |
| rs2565200 | 24 (29.3) | **0.03184** | 106 (45.3) | **0.00000** | **0.01113** | 0.50 (0.29 – 0.86) | **0.01603** |
| rs143948954 | 1 (1.2) | 0.43718 | 7 (3.0) | **0.00924** | 0.37940 | 0.40 (0.05 – 3.30) | 0.63798 |
| rs2435355 | 32 (40.0) | **0.00644** | 72 (30.8) | 0.12127 | 0.12993 | 1.50 (0.89 – 2.54) | 0.16858 |
