# Supplementary material for: RET Variants and Haplotype Analysis in a Cohort of Czech Patients with Hirschsprung Disease
Source: PLoS One. 2014 Jun 4;9(6):e98957. doi: 10.1371/journal.pone.0098957 (PMC4045806; doi:10.1371/journal.pone.0098957)
Supplement: Table S2 — Allelic distribution of single nucleotide polymorphisms in male and female HSCR patients. (DOC) [file pone.0098957.s002.doc]

**Table S2** Allelic distribution of single nucleotide polymorphisms in male and female HSCR patients

|  | **Male cases** (n=121) **vs. male controls** (n=95) | | | | | **Female cases** (n=41) **vs. female controls** (n=110) | | | | |  |
| --- | --- | --- | --- | --- | --- | --- | --- | --- | --- | --- | --- |
| **SNP** | **Cases: Variant** **allele (%)** | **Controls: Variant** **allele (%)** | **p-value (for χ^2^)** | **OR** (**95% CI**) | **p-value (for OR)** | **Cases: Variant** **allele (%)** | **Controls: Variant** **allele (%)** | **p-value (for χ^2^)** | **OR** (**95% CI**) | **p-value (for OR)** | **p-value (male vs. female HSCR)** |
| rs1864410 | 184 (76.0) | 53 (27.9) | **0.00000** | 8.20 (5.32-12.65) | **0.00000** | 49 (59.8) | 60 (27.3) | **0.00000** | 3.96 (2.33-6.74) | **0.00000** | **0.00459** |
| rs2435357 | 185 (76.4) | 54 (28.4) | **0.00000** | 8.17 (5.30-12.60) | **0.00000** | 49 (59.8) | 61 (27.7) | **0.00000** | 3.87 (2.28-6.58) | **0.00000** | **0.00354** |
| rs2506004 | 185 (76.4) | 54 (28.4) | **0.00000** | 8.17 (5.30-12.60) | **0.00000** | 49 (59.8) | 61 (27.7) | **0.00000** | 3.87 (2.28-6.58) | **0.00000** | **0.00354** |
| rs1800858 | 181 (74.8) | 53 (27.9) | **0.00000** | 7.67 (4.99-11.79) | **0.00000** | 50 (61.0) | 59 (26.8) | **0.00000** | 4.26 (2.50-7.28) | **0.00000** | **0.01683** |
| rs1800860 | 62 (26.1) | 67 (35.3) | **0.03905** | 0.65 (0.43-0.98) | 0.05027 | 20 (25.0) | 72 (32.7) | 0.19929 | 0.69 (0.38-1.22) | 0.25346 | 0.85260 |
| rs1799939 | 19 (7.9) | 41 (21.6) | **0.00004** | 0.31 (0.17-0.55) | **0.00008** | 8 (9.8) | 53 (24.1) | **0.00579** | 0.34 (0.15-0.75) | **0.00937** | 0.58963 |
| rs1800861 | 111 (45.9) | 42 (22.3) | **0.00000** | 2.95 (1.92-4.51) | **0.00000** | 30 (36.6) | 47 (21.4) | **0.00695** | 2.12 (1.22-3.69) | **0.01075** | 0.14285 |
| rs111264957 | 3 (1.2) | 6 (3.2) | 0.16587 | 0.38 (0.10-1.56) | 0.29544 | 3 (3.7) | 6 (2.7) | 0.67208 | 1.35 (0.33-5.55) | 0.96583 | 0.16028 |
| rs1800862 | 5 (2.1) | 6 (3.2) | 0.47459 | 0.65 (0.19-2.15) | 0.68374 | 3 (3.7) | 6 (2.7) | 0.67208 | 1.35 (0.33-5.55) | 0.96583 | 0.42193 |
| rs2472737 | 60 (24.8) | 46 (24.2) | 0.88886 | 1.03 (0.66-1.61) | 0.97837 | 22 (26.8) | 53 (24.1) | 0.62424 | 1.16 (0.65-2.06) | 0.73376 | 0.71402 |
| rs1800863 | 20 (8.3) | 41 (21.6) | **0.00008** | 0.33 (0.18-0.58) | **0.00014** | 8 (9.8) | 53 (24.1) | **0.00579** | 0.34 (0.15-0.75) | **0.00937** | 0.67781 |
| rs2565200 | 106 (43.8) | 35 (18.4) | **0.00000** | 3.45 (2.21-5.39) | **0.00000** | 27 (32.9) | 42 (19.1) | **0.01087** | 2.08 (1.18-3.68) | **0.01672** | 0.08362 |
| rs143948954 | 6 (2.5) | 1 (0.5) | 0.11052 | 4.81 (0.57-40.26) | 0.22552 | 2 (2.4) | 1 (0.5) | 0.12196 | 5.48 (0.49-61.21) | 0.37119 | 0.98378 |
| rs2435355 | 81 (33.5) | 46 (24.2) | **0.03599** | 1.57 (1.03-2.41) | **0.04652** | 24 (30.0) | 57 (25.9) | 0.48032 | 1.23 (0.70-2.16) | 0.57633 | 0.56587 |
