# Supplementary material for: RET Variants and Haplotype Analysis in a Cohort of Czech Patients with Hirschsprung Disease
Source: PLoS One. 2014 Jun 4;9(6):e98957. doi: 10.1371/journal.pone.0098957 (PMC4045806; doi:10.1371/journal.pone.0098957)
Supplement: Table S3 — Allelic distribution of single nucleotide polymorphisms considering genotypic distribution of haploblock-haplotypes TTAA and GCCG in HSCR patients and controls. (DOC) [file pone.0098957.s003.doc]

**Table S3** Allelic distribution of single nucleotide polymorphisms considering genotypic distribution of haploblock-haplotypes TTAA and GCCG in HSCR patients and controls

|  | **TTAA/TTAA:**  **Cases** (n=88) **vs. controls** (n=13) | | | | **TTAA/GCCG:**  **Cases** (n=50) **vs. controls** (n=75) | | | | **GCCG/GCCG:**  **Cases** (n=18) **vs. controls** (n=106) | | | |
| --- | --- | --- | --- | --- | --- | --- | --- | --- | --- | --- | --- | --- |
| **SNP** | **Cases: Variant allele (%)** | **Controls: Variant allele (%)** | **OR (95% CI)** | **p-value** | **Cases: Variant allele (%)** | **Controls: Variant allele (%)** | **OR (95% CI)** | **p-value** | **Cases: Variant allele (%)** | **Controls: Variant allele (%)** | **OR (95% CI)** | **p-value** |
| rs1800860 | 40 (23.0) | 4 (15.4) | 1.64 (0.53-5.04) | 0.53576 | 28 (28.6) | 42 (28.0) | 1.03 (0.58-1.81) | 0.96288 | 13 (38.2) | 87 (41.0) | 0.89 (0.42-1.87) | 0.90386 |
| rs1799939 | 1 (0.6) | 0 (0.0) | 0.31 (0.03-3.50) | 0.87562 | 13 (13.0) | 19 (12.7) | 1.03 (0.48-2.19) | 0.90771 | 11 (30.6) | 71 (33.5) | 0.87 (0.41-1.88) | 0.87721 |
| rs1800861 | 99 (56.3) | 13 (50.0) | 1.29 (0.56-2.93) | 0.69865 | 35 (35.0) | 52 (34.7) | 1.01 (0.60-1.73) | 0.93520 | 5 (13.9) | 17 (8.1) | 1.83 (0.63-5.32) | 0.41827 |
| rs111264957 | 0 (0.0) | 0 (0.0) | - | - | 4 (4.0) | 3 (2.0) | 2.04 (0.45-9.32) | 0.58384 | 2 (5.6) | 8 (3.8) | 1.50 (0.31-7.37) | 0.96463 |
| rs1800862 | 0 (0.0) | 0 (0.0) | - | - | 6 (6.0) | 3 (2.0) | 3.13 (0.76-12.81) | 0.18794 | 2 (5.6) | 8 (3.8) | 1.50 (0.31-7.37) | 0.96463 |
| rs2472737 | 55 (31.3) | 12 (46.2) | 0.53 (0.23-1.22) | 0.19931 | 19 (19.0) | 39 (26.0) | 0.67 (0.36-1.24) | 0.25779 | 4 (11.1) | 40 (18.9) | 0.54 (0.18-1.61) | 0.37322 |
| rs1800863 | 2 (1.1) | 0 (0.0) | 0.46 (0.05-4.61) | 0.94868 | 13 (13.0) | 19 (12.7) | 1.03 (0.48-2.19) | 0.90771 | 11 (30.6) | 71 (33.5) | 0.87 (0.41-1.88) | 0.87721 |
| rs2565200 | 98 (55.7) | 13 (50.0) | 1.26 (0.55-2.86) | 0.73959 | 30 (30.0) | 50 (33.3) | 0.86 (0.50-1.48) | 0.67805 | 3 (8.3) | 7 (3.3) | 2.66 (0.66-10.81) | 0.33670 |
| rs143948954 | 6 (3.4) | 1 (3.9) | 0.88 (0.10-7.64) | 0.64506 | 2 (2.0) | 1 (0.7) | 3.04 (0.27-33.99) | 0.72207 | 0 (0.0) | 0 (0.0) | - | - |
| rs2435355 | 67 (38.1) | 10 (38.5) | 0.98 (0.42-2.29) | 0.85892 | 26 (26.5) | 41 (27.3) | 0.96 (0.54-1.71) | 0.99435 | 7 (19.4) | 44 (20.8) | 0.92 (0.38-2.24) | 0.96557 |
