# Supplementary material for: RET Variants and Haplotype Analysis in a Cohort of Czech Patients with Hirschsprung Disease
Source: PLoS One. 2014 Jun 4;9(6):e98957. doi: 10.1371/journal.pone.0098957 (PMC4045806; doi:10.1371/journal.pone.0098957)
Supplement: Table S4 — Allelic distribution of single nucleotide polymorphisms considering genotypic distribution haploblock-haplotypes TTAA and GCCG in patients with long-segment and short-segment form of HSCR. (DOC) [file pone.0098957.s004.doc]

**Table S4** Allelic distribution of single nucleotide polymorphisms considering genotypic distribution haploblock-haplotypes TTAA and GCCG in patients with long-segment and short-segment form of HSCR

|  | **TTAA/TTAA:**  **L-HSCR** (n=19) **vs. S-HSCR** (n=68) | | | | **TTAA/GCCG:**  **L-HSCR** (n=15) **vs. S-HSCR** (n=34) | | | | **GCCG/GCCG:**  **L-HSCR** (n=4) **vs. S-HSCR** (n=12) | | | |
| --- | --- | --- | --- | --- | --- | --- | --- | --- | --- | --- | --- | --- |
| **SNP** | **L-HSCR: Variant** **allele (%)** | **S-HSCR: Variant** **allele (%)** | **OR** (**95% CI**) | **p-value** | **L-HSCR: Variant** **allele (%)** | **S-HSCR: Variant** **allele (%)** | **OR** (**95% CI**) | **p-value** | **L-HSCR: Variant** **allele (%)** | **S-HSCR: Variant** **allele (%)** | **OR** (**95% CI**) | **p-value** |
| rs1800860 | 5 (13.2) | 35 (26.1) | 0.43 (0.16-1.18) | 0.14655 | 9 (30.0) | 19 (28.8) | 1.06 (0.41-2.73) | 0.90360 | 3 (37.5) | 10 (41.7) | 0.84 (0.16-4.35) | 0.83538 |
| rs1799939 | 1 (2.6) | 0 (0.0) | 7.21 (0.64-81.68) | 0.24929 | 3 (10.0) | 10 (14.7) | 0.64 (0.16-2.53) | 0.75664 | 3 (37.5) | 7 (29.2) | 1.46 (0.27-7.82) | 1.00000 |
| rs1800861 | 14 (36.8) | 84 (61.8) | 0.36 (0.17-0.76) | **0.01066** | 12 (40.0) | 21 (30.9) | 1.49 (0.61-3.65) | 0.51676 | 1 (12.5) | 3 (12.5) | 1.00 (0.09-11.24) | 0.53709 |
| rs111264957 | 0 (0.0) | 0 (0.0) | - | - | 2 (6.7) | 1 (1.5) | 4.79 (0.42-54.94) | 0.45928 | 1 (12.5) | 1 (4.2) | 3.29 (0.18-59.60) | 1.00000 |
| rs1800862 | 0 (0.0) | 0 (0.0) | - | - | 3 (10.0) | 2 (2.9) | 3.67 (0.58-23.19) | 0.33425 | 1 (12.5) | 1 (4.2) | 3.29 (0.18-59.60) | 1.00000 |
| rs2472737 | 18 (47.4) | 36 (26.5) | 2.50 (1.19-5.25) | **0.02361** | 6 (20.0) | 13 (19.1) | 1.06 (0.36-3.11) | 0.86078 | 2 (25.0) | 2 (8.3) | 3.67 (0.42-31.73) | 0.53709 |
| rs1800863 | 1 (2.6) | 1 (0.7) | 3.65 (0.22-59.74) | 0.91334 | 3 (10.0) | 10 (14.7) | 0.64 (0.16-2.53) | 0.75664 | 3 (37.5) | 7 (29.2) | 1.46 (0.27-7.82) | 1.00000 |
| rs2565200 | 15 (39.5) | 82 (60.3) | 0.43 (0.21-0.90) | **0.03575** | 9 (30.0) | 20 (29.4) | 1.03 (0.40-2.63) | 0.85614 | 0 (0.0) | 2 (8.3) | 0.85 (0.08-9.30) | 0.64519 |
| rs143948954 | 1 (2.6) | 5 (3.7) | 0.71 (0.08-6.25) | 0.84874 | 0.(0.0) | 2 (2.9) | 0.72 (0.07-7.21) | 0.78759 | 0 (0.0) | 0 (0.0) | - | - |
| rs2435355 | 20 (52.6) | 46 (33.8) | 2.17 (1.05-4.51) | 0.05443 | 6 (21.4) | 20 (29.4) | 0.65 (0.23-1.86) | 0.58411 | 3 (37.5) | 4 (16.7) | 3.00 (0.50-17.95) | 0.45890 |
