# Supplementary material for: RET Variants and Haplotype Analysis in a Cohort of Czech Patients with Hirschsprung Disease
Source: PLoS One. 2014 Jun 4;9(6):e98957. doi: 10.1371/journal.pone.0098957 (PMC4045806; doi:10.1371/journal.pone.0098957)
Supplement: Table S5 — Allelic distribution of single nucleotide polymorphisms considering haplotype TTAA in male and female HSCR patients. (DOC) [file pone.0098957.s005.doc]

**Table S5** Allelic distribution of single nucleotide polymorphisms considering haplotype TTAA in male and female HSCR patients

|  | **TTAA/TTAA:**  **Cases: male** (n=71) **vs. female** (n=17) | | | |
| --- | --- | --- | --- | --- |
| **SNP** | **Male: Variant** **allele (%)** | **Female: Variant** **allele (%)** | **OR** (**95% CI**) | **p-value** |
| rs1800860 | 33 (23.6) | 7 (20.6) | 1.19 (0.47-2.98) | 0.88579 |
| rs1799939 | 1 (0.7) | 0 (0.0) | 0.49 (0.04-5.59) | 0.88427 |
| rs1800861 | 82 (57.8) | 17 (50.0) | 1.37 (0.65-2.89) | 0.53169 |
| rs111264957 | 0 (0.0) | 0 (0.0) | - | - |
| rs1800862 | 0 (0.0) | 0 (0.0) | - | - |
| rs2472737 | 43 (30.3) | 12 (35.3) | 0.80 (0.36-1.75) | 0.71853 |
| rs1800863 | 2 (1.4) | 0 (0.0) | 0.74 (0.08-7.38) | 0.70451 |
| rs2565200 | 81 (57.0) | 17 (50.0) | 1.33 (0.63-2.81) | 0.58210 |
| rs143948954 | 4 (2.8) | 2 (5.9) | 0.46 (0.08-2.64) | 0.71982 |
| rs2435355 | 53 (37.3) | 14 (41.2) | 0.85 (0.40-1.82) | 0.82669 |
